# Serum *O*-glycosylated hepatitis B surface antigen levels in patients with chronic hepatitis B during nucleos(t)ide analog therapy

**DOI:** 10.1186/s12876-022-02352-4

**Published:** 2022-05-31

**Authors:** Ayato Murata, Kiyohiko Angata, Maki Sogabe, Shunsuke Sato, Takafumi Ichida, Hisashi Narimatsu, Takuya Genda

**Affiliations:** 1 Department of Gastroenterology and Hepatology, Juntendo University Shizuoka Hospital, 1129 Nagaoka, Izunokuni-shi, Shizuoka 410-2295 Japan; 2 Research Core for Medical GlycoScience (RCMG) Inc, Tsukuba, Ibaraki Japan; 3 Department of Hepatology, East Shonan Clinic, Chigasaki, Kanagawa Japan

**Keywords:** Hepatitis B virus, Hepatitis B surface antigen, *O*-Glycan, Pregenomic RNA, Virion

## Abstract

**Background:**

Serum hepatitis B surface antigen (HBsAg) is a component of both hepatitis B virus (HBV) virions and non-infectious subviral particles (SVPs). Recently, *O*-glycosylation of the PreS2 domain of middle HBsAg protein has been identified as a distinct characteristic of genotype C HBV virions versus SVPs. This study aimed to evaluate serum *O*-glycosylated HBsAg levels in patients with chronic hepatitis B (CHB) treated with nucleos(t)ide analogs (NAs).

**Methods:**

Forty-seven treatment-naïve patients with genotype C CHB were retrospectively enrolled. Serum *O*-glycosylated HBsAg levels at baseline and after 48 weeks of NA therapy were quantified by immunoassay using a monoclonal antibody against the *O*-glycosylated PreS2 domain of middle HBsAg, and their correlations with conventional HBV marker levels were analyzed.

**Results:**

At baseline, the serum *O*-glycosylated HBsAg levels were significantly correlated with the HBV DNA (*P* = 0.004), HBsAg (*P* = 0.005), and hepatitis B-core related antigen (HBcrAg, *P* = 0.001) levels. Both HBV DNA and *O*-glycosylated HBsAg levels were decreased after 48 weeks of NA therapy. The significant correlation of the *O*-glycosylated HBsAg level with the HBsAg or HBcrAg level was lost in patients who achieved undetectable HBV DNA (HBsAg, *P* = 0.429; HBcrAg, *P* = 0.065). Immunoprecipitation assays demonstrated that HBV DNA and RNA were detected in the *O*-glycosylated HBsAg-binding serum fraction, and the proportion of HBV RNA increased during NA therapy (*P* = 0.048).

**Conclusion:**

Serum *O*-glycosylated HBsAg levels change during NA therapy and may reflect combined levels of serum HBV DNA and RNA virions. An *O*-glycosylated HBsAg-based immunoassay may provide a novel means to monitor viral kinetics during NA therapy.

## Background

Persistent hepatitis B virus (HBV) infection is a major global health problem, and an estimated 291 million individuals worldwide are HBV carriers. Among them, approximately 1 million people die annually due to progression to decompensated cirrhosis and/or development of hepatocellular carcinoma [1, 2]. Despite recent advances in antiviral agents for HBV, such as nucleos(t)ide analogs (NAs), the clinical course of chronic hepatitis B (CHB) is complex and the response to antiviral therapy varies among individuals [3]. Therefore, it is important to monitor viral kinetics, and numerous HBV markers are used in clinical practice [4].

HBV virions are enveloped particles with a diameter of approximately 42 nm (Dane particle). The envelope is formed by three viral surface glycoproteins: large hepatitis B surface antigen (L-HBsAg), middle HBsAg (M-HBsAg), and small HBsAg (S-HBsAg), which surround the viral nucleocapsid. Structurally, the amino-terminal preS1 domain is exclusively present in L-HBsAg and the PreS2 domain is present in both L- and M-HBsAg, whereas the S domain is common to L-, M-, and S-HBsAg [5]. Besides virions, large amounts of non-infectious subviral particles (SVPs) in the form of filaments or spheres are found in the serum of HBV-positive patients. SVPs lack viral nucleic acids and are composed of identical viral surface glycoproteins, L-, M-, and S-HBsAg, although the glycoprotein composition differs from that of virions. Since the virions are essential for HBV infection, monitoring HBV virions rather than SVPs provides important information for the assessment of CHB patients.

HBsAg has long served as a qualitative serological marker for the diagnosis of HBV infection. Quantitative HBsAg assay has demonstrated that serum HBsAg levels are correlated with serum HBV DNA levels and intrahepatic covalently closed circular DNA (cccDNA) levels, and show prognostic significance [6]. However, currently available HBsAg assays cannot distinguish between HBV virions and SVPs. Recently, *O*-glycosylation of the PreS2 domain of M-HBsAg was identified as a distinct characteristic of genotype C HBV virions via a glycan-based immunoassay, and a recombinant antibody that specifically recognizes *O*-glycosylated M-HBsAg (anti-Glyco-PreS2 antibody) was developed [7, 8]. In this study, we developed a quantitative immunoassay for *O*-glycosylated HBsAg using anti-Glyco-PreS2 antibody and analyzed changes in serum *O*-glycosylated HBsAg levels in patients with CHB during NA therapy.

## Methods

### Patients and routine laboratory investigations

The study cohort consisted of 47 patients who were persistently infected with genotype C HBV and started initial NA therapy at Juntendo University Shizuoka Hospital. NA therapy was indicated for CHB with an HBV DNA level of at least 3.3 log IU/mL and alanine aminotransferase (ALT) level of at least 31 U/L, and for liver cirrhosis with HBV DNA positive regardless of the ALT level according to the guidelines of the Japanese Society of Hepatology [9]. The eligible patients were confirmed as being HBsAg-positive for > 6 months, for whom serum samples were available prior to and at 48 weeks after the initiation of treatment. The exclusion criteria were (1) positivity for anti-hepatitis C virus antibodies, (2) history or serologic evidence of any other chronic liver diseases (i.e., autoimmune hepatitis, primary biliary cholangitis, and hemochromatosis). The medical records of all participants were retrospectively reviewed to obtain clinical data. All routine conventional HBV markers were collected immediately before the first NA treatment and at 48 weeks after treatment initiation. Serum HBV DNA levels were determined using a COBAS TaqMan HBV test v2.0 (Roche Diagnostics, Branchburg, NJ, USA), which has a dynamic range of 1.3–8.2 log IU/mL. Serum levels of HBsAg (dynamic range, − 1.3 to 5.1 log IU/mL) and hepatitis B core-related antigen (HBcrAg, dynamic range, 2.9–7.0 log U/mL) were quantified using commercial chemiluminescent enzyme immunoassay kits. Serum hepatitis B e antigen (HBeAg) was detected using a commercial chemiluminescent enzyme immunoassay kit and positivity was defined as levels ≥ 1.0 cut-off index.

### Measurement of serum *O*-glycosylated HBsAg levels by enzyme-linked immunoassay (ELISA) using anti-Glyco-PreS2 antibody

A monoclonal antibody to *O*-glycosylated PreS2 in M-HBsAg (anti-Glyco-PreS2) and M-HBsAg as a standard protein was prepared as reported previously [8]. An Immobilizer Amino 96-well plate (Thermo Fisher Scientific, Waltham, MA, USA) was coated with anti-Glyco-PreS2 (0.5 µg/well) at room temperature for 3 h and blocked with sucrose Tris-buffered saline containing Tween-20 (TBS-T; 5% sucrose, 5% Tween-20, 50 mM Tris–Cl pH 8.0, 0.15 M NaCl, 0.1% NaN_3_). Patient serum samples (2 µL) and negative control (2 µL of pooled normal human serum, Cosmo Bio, Tokyo, Japan) were diluted with 100 µL of dilution buffer (3% bovine serum albumin, 0.1% Tween-20, 50 mM Tris pH 8.0, 0.15 M NaCl, 0.1% NaN_3_). ELISA standard proteins (recombinant M-HBsAg for calibration) were serially diluted with 2% normal human serum/dilution buffer to generate a standard curve. The patient serum samples (100 µL) were added into each well of the plate, which was then gently mixed for 2 h at room temperature. After washing with phosphate-buffered saline containing 0.05% Tween-20 (PBS-T), 100 µL of anti-Glyco-PreS2 (1 µg/mL) labeled with biotin (Dojindo Laboratories, Kumamoto, Japan) was added into each well and the plate was incubated at room temperature for 1.5 h. Then, 100 µL of streptavidin–horseradish peroxidase (HRP) (0.05 µg/mL PBS-T, Jackson ImmunoResearch Laboratories, West Grove, PA, USA) and 100 µL of HRP substrate (1Step Ultra TMB-ELISA, Thermo Fisher Scientific) were added to each well. After reaction, the absorbance at 450 nm was measured using an ELISA plate reader (Bio-Rad, Hercules, CA, USA). Serum *O*-glycosylated M-HBsAg levels were calculated based on the standard curve and dilution factor.

### Immunoprecipitation of HBV particles with anti-Glyco-PreS2 and quantification of HBV DNA and RNA

One microgram biotinylated anti-Glyco-PreS2 was mixed with 10 µL of streptavidin-conjugated magnetic beads (Dynabeads MyOne Streptavidin T1, Thermo Fisher Scientific) in PBS-T containing 0.1% Tween-20 at 4 °C for 30 min. Serum samples of patients infected with HBV (10 µL) were mixed with the antibody-bound beads in 100 µL of PBS-T at 4 °C overnight. The beads were collected using a magnetic stand to separate the immunoprecipitated (IP) and supernatant fractions. Quantitative (q)PCR amplification of HBV DNA and RNA in the IP fraction was conducted as reported previously [7, 10].

Briefly, to quantify viral DNA, viral DNA was isolated from the IP fraction using a QIAamp DNA Mini Kit (Qiagen, Hilden, Germany). The viral DNA was amplified using Taq DNA polymerase (Fast SYBR Green Master Mix, Thermo Fisher Scientific) and primers targeting the S-HBsAg sequence, HBV-SF2 (5′-CTTCATCCTGCTGCTATGCCT-3′ [nt 406–426]) and HBV-SR2 (5′-AAAGCCCAGGATGATGGGAT-3′ [nt 627–608]) [7], on an Applied Biosystems 7500 Real-Time PCR system (Thermo Fisher Scientific). The DNA was denatured at 95 °C for 10 min and amplified in 45 cycles (95 °C for 15 s and 60 °C for 60 s). cDNA encoding M-HBsAg (GenBank ID: AB246345) was used as standard DNA.

To quantify HBV RNA, RNA was isolated from the IP fraction using an RNeasy Plus Mini Kit (Qiagen). The RNA was treated with DNase (Promega, Madison, WI, USA) and reverse-transcribed using SuperScript IV (Thermo Fisher Scientific) and an HBV-specific primer, H-RT (5′-GACGTTGTAAAACGACGGCCAGGCCTCAAGGTCGGTCGTTGAC-3′), which contains an M13 phage sequence (M13-F, 5′-GACGTTGTAAAACGACGGCCAG-3′) and an HBV-specific sequence (5′-GCCTCAAGGTCGGTCGTTGAC-3′ [nt 1702–1682]) [9]. Then, the cDNA was subjected to qPCR for amplification and quantification as described above, using the M13-F primer and HBV-specific primer H-F (5′-CTGTGCCTTCTCATCTGCCG-3′ [nt 1553–1572]). The proportion of HBV RNA in the IP fraction was calculated as the amount of HBV RNA divided by the sum of the amounts of HBV RNA and DNA.

### Statistical analyses

Statistical analyses were conducted using the Mann–Whitney *U* test, Spearman rank correlation test, and Wilcoxon’s signed rank test in IBM SPSS Statistics 24 (SPSS Inc., Chicago, IL, USA). The distribution of continuous variables was also analyzed using the Shapiro–Wilk test. A *P* value < 0.05 was considered statistically significant.

## Results

### Baseline characteristics of patients

The baseline characteristics of the 47 patients included in this study are summarized in Table 1. There were31 men and 16 women, and their median age was 55 (range, 27–80) years. The NA therapy was initiated as follows: 43 (91.4%) patients received entecavir monotherapy, 3 (6.4%) patients received lamivudine monotherapy, and 1 (2.1%) patient received tenofovir disoproxil fumarate monotherapy.

**Table 1:** Patients’ characteristics at baseline, by HBeAg status

Characteristics	All patients (n = 47)	HBeAg positive (n = 25)	HBeAg negative (n = 22)
Age (years)	55 (27–80)	55 (27–80)	57 (35–80)
Males	31 (70.0)	15 (60.0)	16 (72.7)
Albumin (g/dL)	4.1 (2.4–4.6)	4.0 (2.4–4.6)	4.1 (3.5–4.6)
ALT (IU/L)	58 (14–538)	72 (29–522)	45 (14–538)
Total bilirubin (mg/dL)	0.8 (0.3–3.7)	0.8 (0.4–2.4)	0.9 (0.3–3.7)
Cirrhosis	14 (29.8)	6 (24.0)	8 (36.4)
HBV-DNA (log IU/mL)	5.7 (1.3–8.2)	6.8 (1.7–8.2)	4.4 (1.3–7.7)
HBsAg (log IU/mL)	3.3 (− 0.1 to 4.8)	3.5 (2.7–4.8)	3.3 (− 0.1 to 4.0)
HBcrAg (log U/mL)	5.7 (2.9–7.0)	7.0 (5.4–7.0)	4.1 (2.9–6.4)

Data are expressed as median (range) or number (%)

*ALT* alanine aminotransferase, *HBcrAg* hepatitis B core-related antigen, *HBeAg* hepatitis B e antigen, *HBsAg* hepatitis B surface antigen, *HBV* hepatitis B virus

### Baseline correlations between conventional HBV marker levels and serum *O*-glycosylated HBsAg levels

**Fig. 1:**
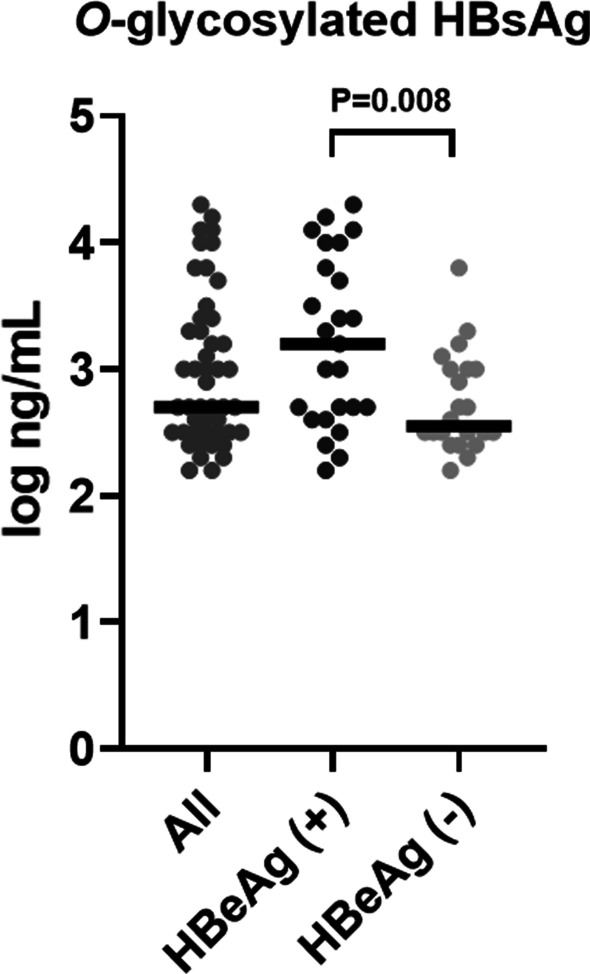
Distribution of baseline *O*-glycosylated HBsAg serum levels in the study cohort. The bar in each scatter plot represents the median value. Results for HBeAg-positive and -negative patients were compared using the Mann–Whitney *U*-test. *HBeAg* hepatitis B e antigen, *HBsAg* hepatitis B surface antigen

At baseline, the median serum HBsAg level was 3.3 log IU/mL (range, –0.1–4.8 log IU/mL), the median serum HBV DNA level was 6.5 log IU/mL (range, 1.3–8.1 log IU/mL), and the median HBcrAg level was 5.7 log U/mL (range, 2.9–7.0 log U/mL). HBeAg positivity was observed in 25 out of 47 patients (53.2%). The distribution of the baseline serum *O*-glycosylated HBsAg levels is shown in Fig. 1. The median serum *O*-glycosylated HBsAg level was 2.7 log ng/mL (range, 2.2–4.3 log ng/mL) and was significantly higher in HBeAg-positive patients than in HBeAg-negative patients (3.2 vs. 2.6 log ng/mL, *P* = 0.008).

**Fig. 2:**
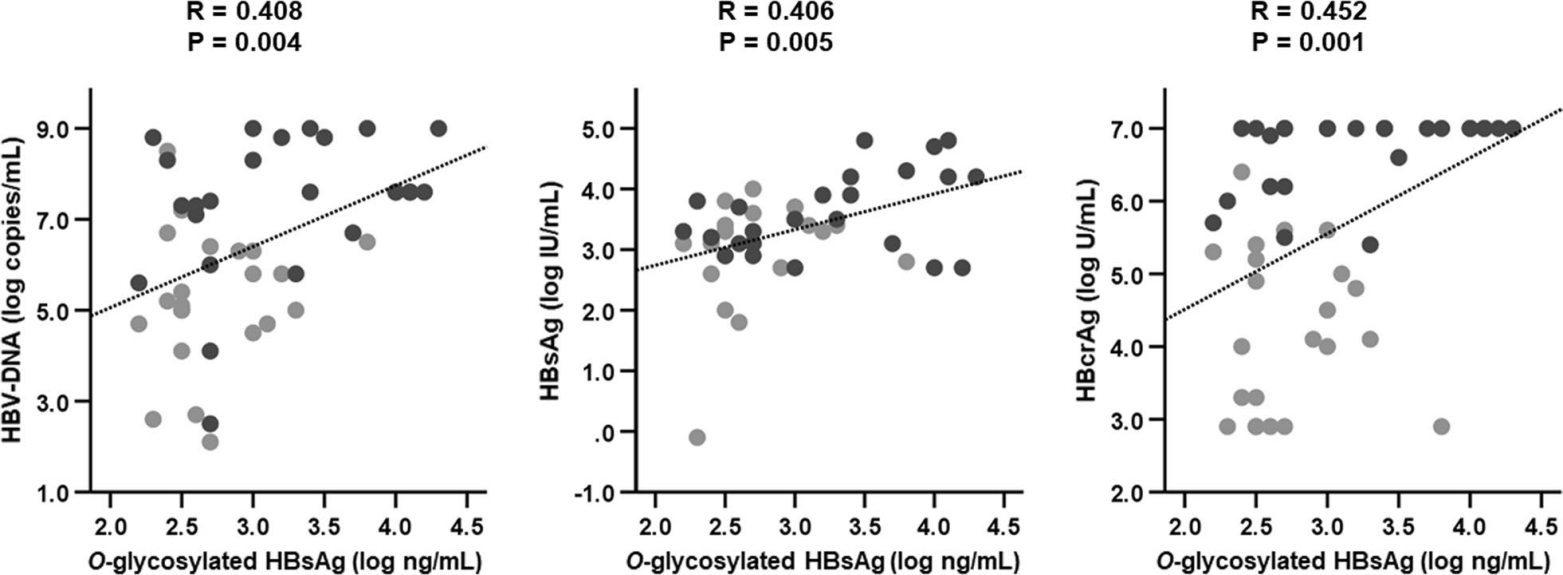
Relationships between *O*-glycosylated HBsAg levels and conventional HBV marker levels at baseline. Data were analyzed using Spearman’s rank correlation coefficient. Light gray circles represent HBeAg-negative patients, dark gray circles represent HBeAg-positive patients. *HBcrAg* hepatitis B core-related antigen, *HBeAg* hepatitis B e antigen, *HBsAg* hepatitis B surface antigen, *HBV* hepatitis B virus

Figure 2 shows the baseline correlations between conventional HBV marker levels and *O*-glycosylated HBsAg levels. Serum *O*-glycosylated HBsAg levels were significantly correlated with HBV DNA (*P* = 0.004), HBsAg (*P* = 0.005), and HBcrAg (*P* = 0.001) levels.

### Changes in HBV marker levels and *O*-glycosylated HBsAg levels during NA therapy

**Fig. 3:**
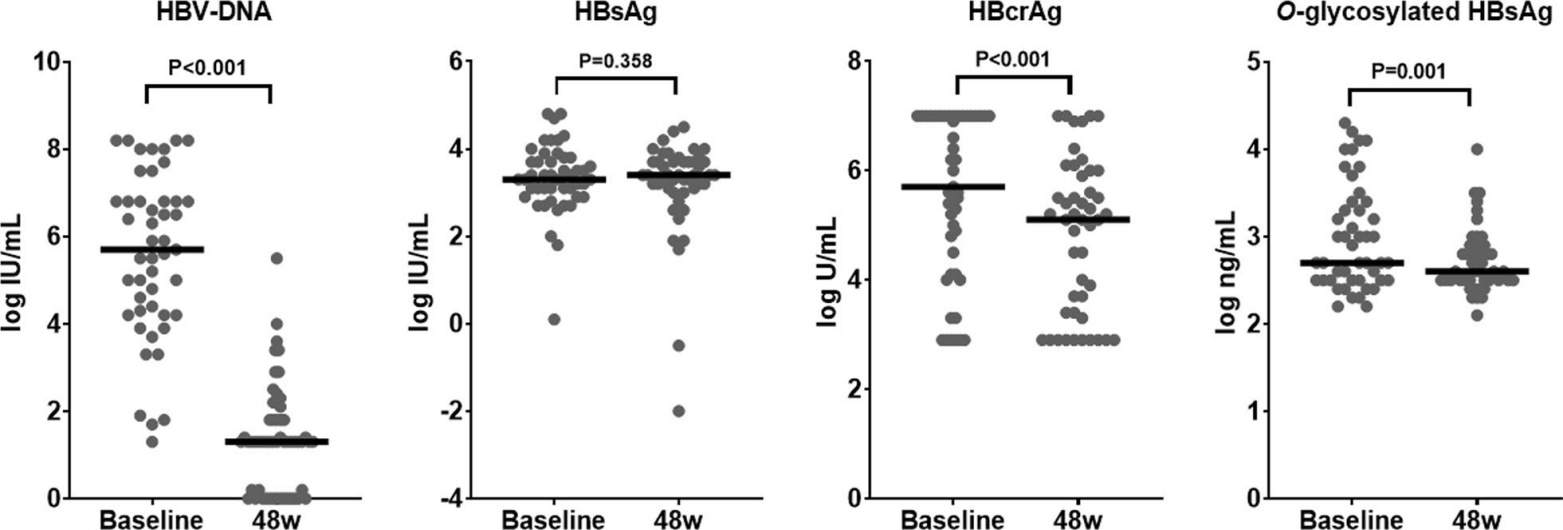
Comparison of HBV marker levels at baseline and after 48 weeks of NA therapy. The bar in each scatter plot represents the median value. Data were analyzed using Wilcoxon’s signed rank test. *HBcrAg* hepatitis B core-related antigen, *HBsAg* hepatitis B surface antigen, *HBV* hepatitis B virus, *NAs* nucleos(t)ide analog.

The median serum HBV DNA level significantly decreased from 5.7 log IU /mL at baseline to 1.3 log IU /mL after 48 weeks of NA therapy (*P* < 0.001, Fig. 3), and 14 (21.3%) patients reached undetectable HBV DNA at 48 weeks. Both serum HBcrAg and *O*-glycosylated HBsAg levels significantly decreased from baseline to 48 weeks (HBcrAg, from 5.7 to 5.1 log U/L, *P* < 0.001; *O*-glycosylated HBsAg, from 2.7 to 2.6 log ng/mL, *P* = 0.001). There were no significant differences in serum HBsAg levels between the two time points (3.3 IU/mL at baseline vs. 3.4 log IU/mL at 48 weeks, *P* = 0.358).

**Fig. 4:**
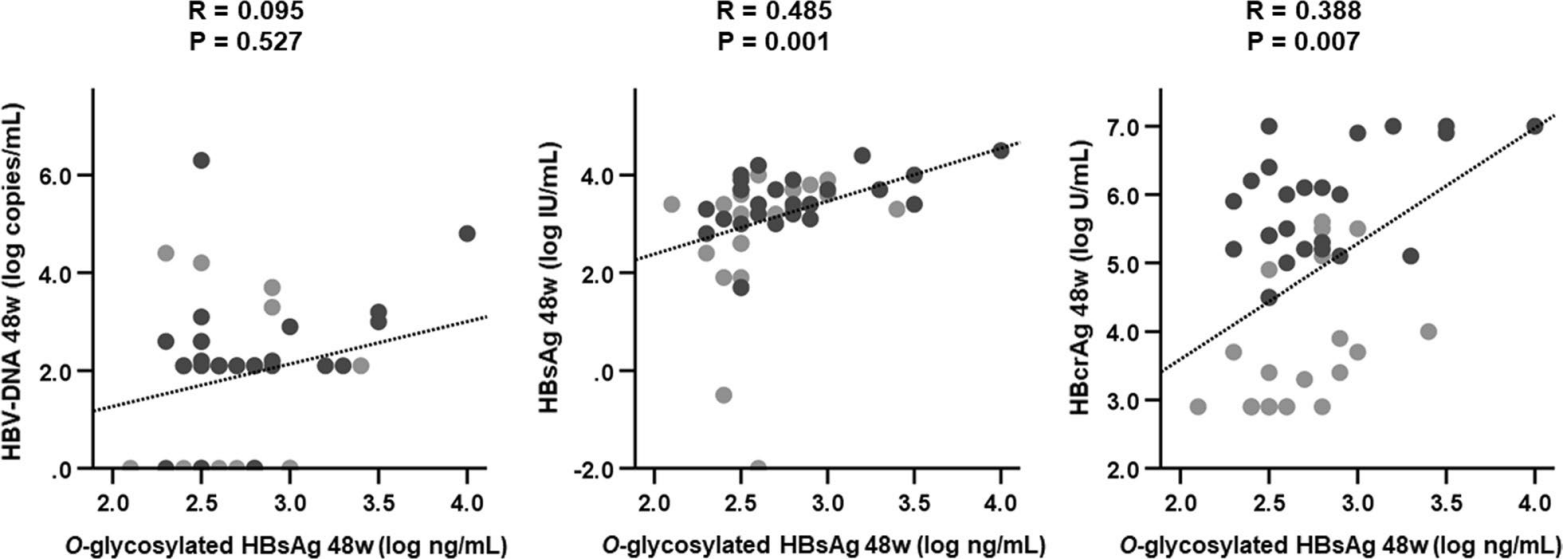
Relationships between *O*-glycosylated HBsAg levels and conventional HBV marker levels after 48 weeks of NA therapy. Data were analyzed using Spearman’s rank correlation coefficient. Light gray circles represent HBeAg-negative patients, dark gray circles represent HBeAg-positive patients. *HBcrAg* hepatitis B core-related antigen, *HBeAg* hepatitis B e antigen, *HBsAg* hepatitis B surface antigen, *HBV* hepatitis B virus, *NA* nucleos(t)ide analog

**Fig. 5:**
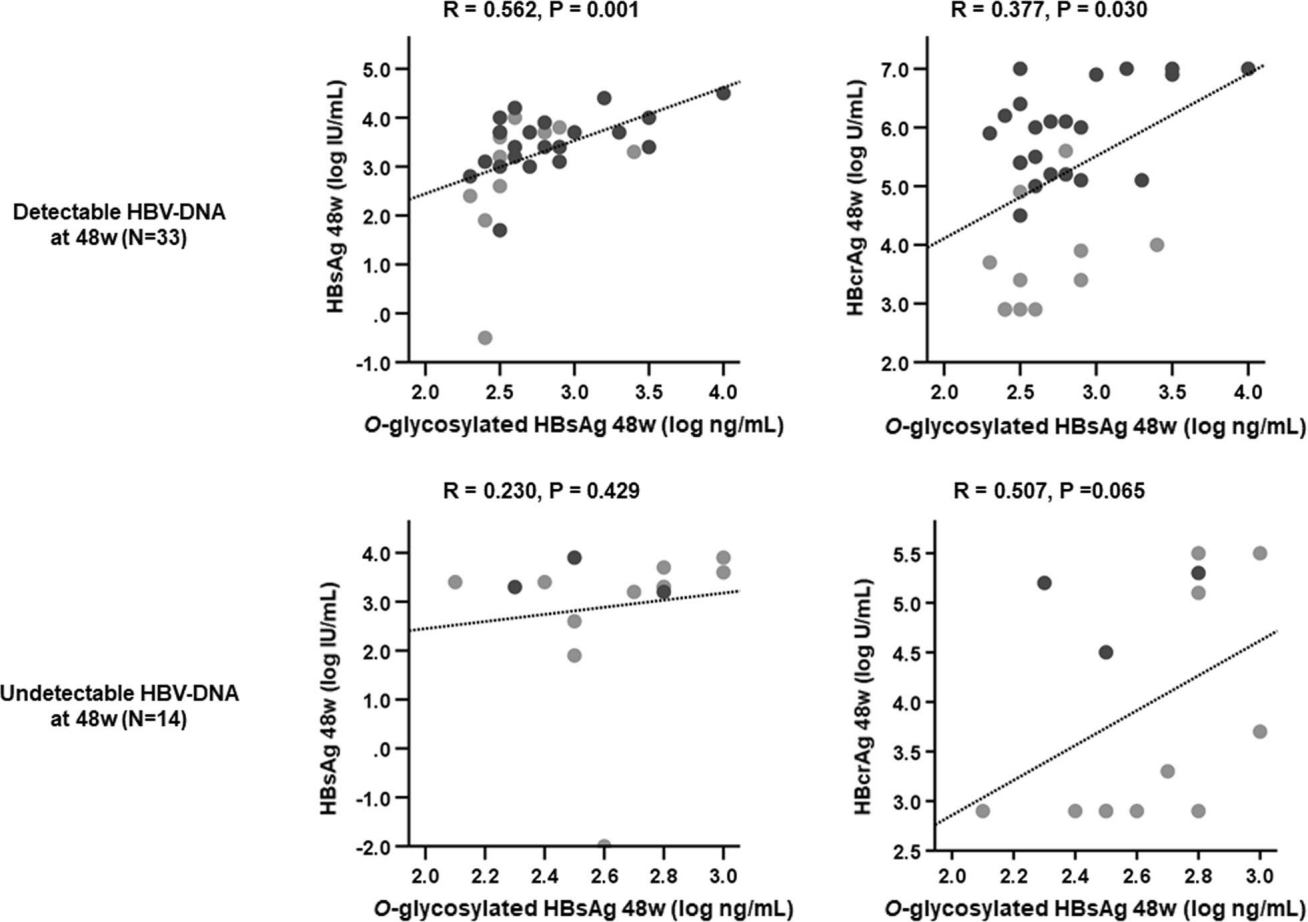
Correlations between *O*-glycosylated HBsAg levels and HBsAg or HBcrAg levels after 48 weeks of NA therapy, stratified by HBV DNA levels at 48 weeks. Data were analyzed using Spearman’s rank correlation coefficient. Light gray circles represent HBeAg-negative patients, dark gray circles represent HBeAg-positive patients. *HBcrAg* hepatitis B core-related antigen, *HBeAg* hepatitis B e antigen, *HBsAg* hepatitis B surface antigen, *NA* nucleos(t)ide analog

Figure 4 shows the correlations between conventional HBV marker levels and *O*-glycosylated HBsAg levels at 48 weeks. The significant correlation between serum *O*-glycosylated HBsAg levels and HBV DNA levels was lost at 48 weeks (*P* = 0.527), but those with HBsAg and HBcrAg levels remained (HBsAg, *P* = 0.001; HBcrAg, *P* = 0.007). Figure 5 illustrates the correlations between *O*-glycosylated HBsAg levels and HBsAg and HBcrAg levels at 48 weeks after stratification by the HBV DNA levels. In 33 patients with detectable HBV DNA in serum at 48 weeks, *O*-glycosylated HBsAg levels remained significantly correlated with HBsAg and HBcrAg levels (HBsAg, *P* = 0.001; HBcrAg, *P* = 0.030), whereas in 14 patients with undetectable HBV DNA, these correlations were lost (HBsAg, *P* = 0.429; HBcrAg, *P* = 0.065).

### HBV DNA and RNA quantification in the *O*-glycosylated HBsAg-binding fraction

**Fig. 6:**
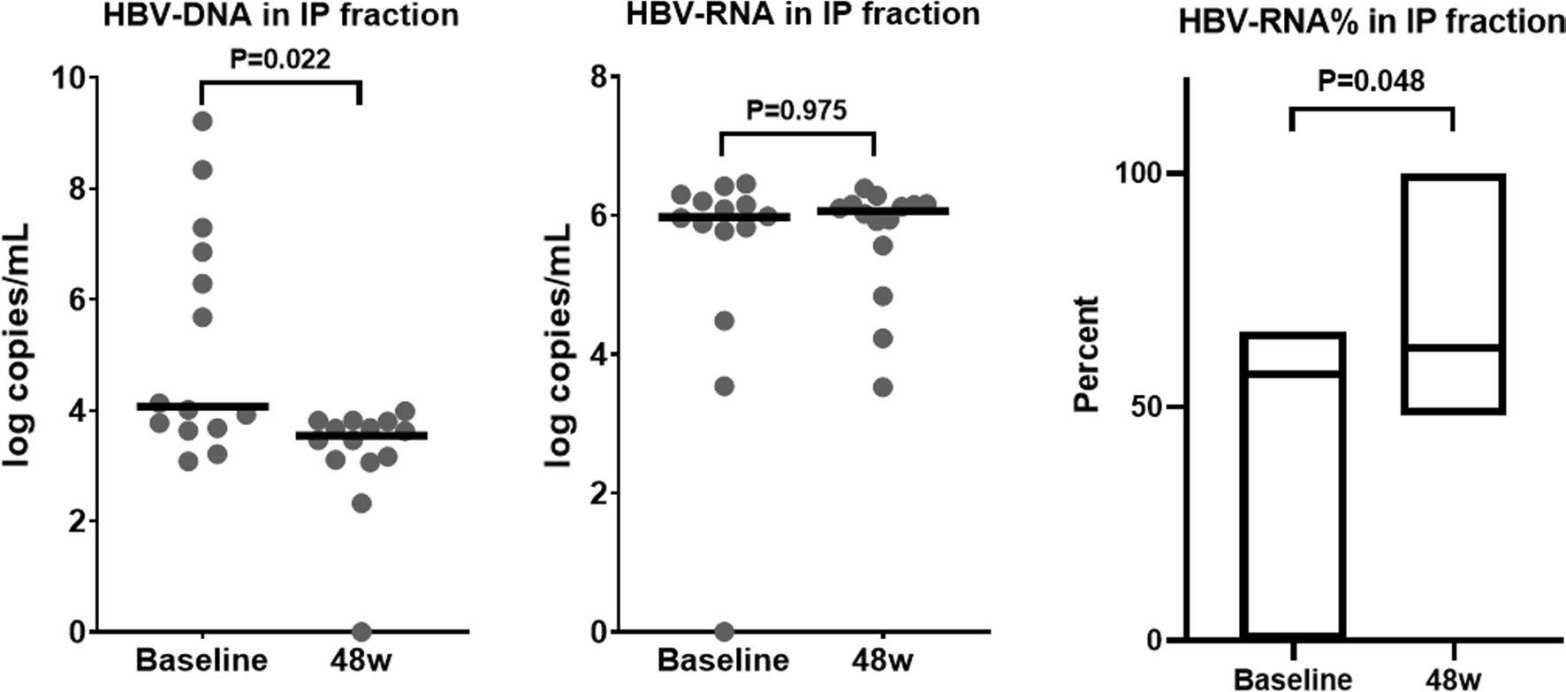
Quantification of serum HBV DNA and RNA levels, and proportion of HBV RNA in the *O*-glycosylated HBsAg-binding fraction in patients with undetectable HBV DNA after 48 weeks of nucleos(t)ide analog therapy. The bars in the scatter plots represent the median values. In the floating boxes, the box length represents the range and the horizontal line within the box represents the median value. Results at baseline and at 48 weeks were compared using Wilcoxon’s signed rank test. *HBcrAg* hepatitis B core-related antigen, *HBsAg* hepatitis B surface antigen, *HBV* hepatitis B virus, *IP* immunoprecipitate

In the 14 patients with undetectable serum HBV DNA at 48 weeks, the *O*-glycosylated HBsAg-binding fraction was extracted using immunoprecipitation and the HBV DNA and RNA levels were determined (Fig. 6). At baseline, both HBV DNA and RNA were detected in the precipitate, which reflected *O*-glycosylated HBsAg-binding fraction. At 48 weeks, despite undetectable serum HBV DNA, HBV DNA in the precipitate remained detectable in 13 patients, and became undetectable in just one patient. However, at 48 weeks, the median HBV DNA level in the precipitate was significantly decreased (from 4.07 to 3.54 log copies/mL, *P* = 0.022), whereas the median HBV RNA level in the precipitate did not show a significant difference (5.98 vs. 6.07 log copies/mL, *P* = 0.975). The median proportion of HBV RNA in the precipitate significantly increased from 56.9% at baseline to 62.7% at 48 weeks (*P* = 0.048).

## Discussion

The PreS2 domain of M-HBsAg from genotype C HBV is partially *O*-glycosylated at the threonine residue at site 37 [11]. Recently, a lectin-fractionation method using jacalin, which recognizes *O*-glycan, was used to enrich HBV DNA particles from patient sera [7]. In addition, antibody against *O*-glycosylated preS2 in M-HBsAg showed a higher efficacy in preventing HBV infection of hepatocytes than human hepatitis B immunoglobulin [8]. Thus, *O*-glycosylated M-HBsAg was identified as one of the primary components of HBV DNA-containing virus particles, and may become a useful tool to discriminate between HBV viral particles and excess amounts of SVPs.

In this study, we developed an immunoassay using a specific antibody against *O*-glycosylated preS2 in M-HBsAg and measured *O*-glycosylated HBsAg serum levels during NA therapy. At baseline, *O*-glycosylated HBsAg levels correlated well with not only HBV DNA, but also total HBsAg and HBcrAg levels. After 48 weeks of NA therapy, serum HBV DNA levels were significantly decreased as NA effectively suppressed the reverse transcription of HBV pregenomic RNA to HBV DNA. This should translate in a decline in the amount of serum HBV virions, which are viral nucleocapsids enveloped by viral surface glycoproteins such as HBsAg, during NA therapy. Indeed, *O*-glycosylated HBsAg serum levels were significantly decreased after 48 weeks of NA therapy. However, the decrease in *O*-glycosylated HBsAg levels was smaller than that in HBV DNA levels. Although the median HBV DNA level decreased from 5.7 to 1.3 log IU/mL and approximately 20% of patients achieved undetectable HBV DNA, the median *O*-glycosylated HBsAg level decreased by only 0.1 log ng/mL. Furthermore, *O*-glycosylated HBsAg remained detectable even in patients who achieved undetectable HBV DNA after 48 weeks of NA therapy. One possible reason is that the level of remnant HBV DNA virions was below the qualitative limit of detection of the commercial HBV DNA assay. Indeed, the existence of infectious HBV virions in serum has been demonstrated in patients in whom the HBV DNA level decreased below the lower limit of quantification after NA therapy [12]. In addition, our immunoprecipitation assay demonstrated the existence of quantifiable HBV DNA in the concentrated *O*-glycosylated HBsAg-binding fraction. However, this explanation seems to be insufficient because the decreases in serum *O*-glycosylated HBsAg levels were demonstrably smaller than those in serum HBV DNA levels. Another probable reason is the existence of HBV RNA virions in the sera. It has been recently reported that HBV RNA virions are abundantly present in sera of CHB patients and significantly increase after NA therapy [10]. We found that not only HBV DNA, but also HBV RNA was detectable in the *O*-glycosylated HBsAg-binding fraction. This result suggests that *O*-glycosylated HBsAg is a component both of HBV DNA and RNA virions, and the serum *O*-glycosylated HBsAg level reflects combined HBV DNA and RNA virion levels. Further analysis of the correlation between the combined levels of serum HBV DNA and RNA and serum *O-*glycosylated HBsAg level might provide more insights. Interestingly, HBV RNA was also detectable in the supernatant fraction in the immunoprecipitation assay (data not shown). This observation suggests that a part of HBV RNA exists in an unenveloped form in patients’ serum.

Currently, oral administration of NAs is the most popular treatment strategy for patients with CHB because of the excellent virologic efficacy and safety profile of NAs. Long-term administration of NAs suppresses HBV replication in most patients, resulting in biochemical remission and histological improvement, including the regression of fibrosis and cirrhosis [13, 14]. However, HBV infection cannot be completely eliminated because of the persistence of intrahepatic cccDNA [15]. Measuring the intrahepatic cccDNA concentration would be the most direct way to assess the replication-competent viral reservoir. However, there are limitations, including the need for liver biopsy and the lack of a standardized method to quantify cccDNA. Recently, serum HBV RNA has been suggested to be useful for monitoring residual transcriptional activities and as a surrogate marker for cccDNA and a possible clinical predictor for NA discontinuation without viral rebound [10, 16]. Our findings indicate that the *O*-glycosylated HBsAg serum level reflects the combined levels of HBV DNA and RNA virions, and in virally suppressed patients with low detectable HBV DNA during NA therapy, it predominantly reflects the amount of RNA virions. Thus, serum *O*-glycosylated HBsAg may be a conventional immunoassay target for monitoring viral kinetics reflecting HBV RNA virion levels and a surrogate marker for cccDNA during NA therapy. Even though serum *O-*glycosylated HBsAg was quantified only in patients with genotype C HBV infection, and not with the other HBV genotypes, it might be a potential biomarker for monitoring safe discontinuation of NA therapy similar to the serum HBV RNA [10]. Furthermore, it might be applicated to predict HBsAg seroclearance. Interestingly, in patients with undetectable HBV DNA after 48 weeks of therapy, the *O*-glycosylated HBsAg level no longer correlated with the total HBsAg and HBcrAg levels, although both HBsAg and HBcrAg have been suggested as potential surrogate markers for cccDNA [17, 18]. The reason for this phenomenon is not fully understood, and further study is needed to determine the clinical usefulness of the serum *O*-glycosylated HBsAg level in comparison with total HBsAg and HBcrAg levels.

The main limitation of this study is it was retrospective single-center analysis of a small number of patients. These factors are associated with risks of bias, and they might decrease the power of the statistical findings. Therefore, a large-scale prospective study will be required to validate our results.

In conclusion, the serum *O*-glycosylated HBsAg level can be used to evaluate serum HBV virion levels through conventional immunoassay and may be a novel potential biomarker of viral kinetics, especially in patients receiving NA therapy. Especially, its usefulness for monitoring safe discontinuation of NA therapy and predicting HBsAg seroclearance requires further research.

## Data Availability

The data that support the findings of this study are available from the corresponding author upon reasonable request.

## Abbreviations

ALT: Alanine aminotransferase
cccDNA: Covalently closed circular DNA
CHB: Chronic hepatitis B
HBcrAg: Hepatitis B core-related antigen
HBeAg: Hepatitis B e antigen
HBsAg: Hepatitis B surface antigen
HBV: Hepatitis B virus
HRP: Horseradish peroxidase
IP: Immunoprecipitate
NAs: Nucleos(t)ide analogs
PBS: Phosphate-buffered saline
SVPs: Subviral particles
TBS: Tris-buffered saline
